# Social Company Disrupts Fear Memory Renewal: Evidence From Two Rodent Studies

**DOI:** 10.3389/fnins.2018.00565

**Published:** 2018-08-17

**Authors:** Jiajin Yuan, Minmin Yan, Yin Xu, Weihai Chen, Xiaqing Wang

**Affiliations:** ^1^College of Education Science, Chengdu University, Chengdu, China; ^2^Key Laboratory of Cognition and Personality of Ministry of Education, Faculty of Psychology, Southwest University, Chongqing, China; ^3^School of Sociology, China University of Political Science and Law, Beijing, China

**Keywords:** social company, social buffering effect, fear conditioning, renewal, context updating, extinction

## Abstract

Renewal of fear outside treatment context is a challenge for behavioral therapies. Prior studies suggest a social buffering effect that fear response is attenuated in the presence of social company. However, few studies have examined the role of social company in reducing fear renewal. Here, we used a Pavlovian fear conditioning procedure including acquisition, extinction and test stages to examine social buffering effect on fear memory renewal in male rats. The test context was manipulated to be either different from the extinction one in ABC model, or same as that in ACC model. All conditioned subjects underwent extinction individually in Experiment 1 but with a partner in Experiment 2. In test, both experiments manipulated social company (alone vs. accompanied) and context (ABC vs. ACC). Experiment 1 showed more freezing in ABC than in ACC model during the test-alone condition, indicating a fear renewal effect which, however, was absent during the test-accompanied condition. Also, accompanied subjects showed less freezing compared to alone subjects in the ABC model. In Experiment 2, animals showed a similar freezing in ABC and ACC models despite being tested alone, implying that social company offered at extinction disrupted fear renewal. Again, we observed reduced freezing in accompanied relative to alone subjects in the test. These results suggest that social company is effective in disrupting fear renewal after leaving treatment context.

## Introduction

Posttraumatic stress disorder (PTSD) is characterized by the intrusive flashback of fear memories or difficulty in fear memory extinction (Jovanovic and Ressler, 2010). Exposure therapy, which is based on extinction theory, aims to treat PTSD patients through repeated presence of trauma-related stimuli in the absence of real threats (Mark and Lovell, 1998). However, approximately 30–50% of patients experience renewal of anxiety symptom when the therapy is finished and patients leave the therapeutic context (Choy et al., 2007). Thus, treatments do not completely erase the original fear memory, but instead it forms context-dependent learning. In other words, the extinguished fear memory tends to renew when fear-related stimulus is presented outside of the extinction context (Bouton and Bolles, 1979). Accordingly, fear memory renewal is defined as the recovery of an extinguished fear response when test occurs in a novel context different from that of extinction (Boschen et al., 2009; Polack et al., 2013).

To simulate PTSD and explore its therapies in the laboratory setting, the Pavlovian fear conditioning paradigm is often used with animal models (Herry et al., 2010; Goode and Maren, 2014). In the Pavlovian fear conditioning paradigm, exposing an animal to the pair of a neutral conditioned stimulus (CS), such as a light or white noise, with an unconditioned stimulus (US), such as a footshock, will lead to a conditioned fear response (CR) when the animal receives the CS alone. The typical renewal procedure includes three stages, that is, the acquisition (presentation of the CS-US), extinction (presentation of the CS alone), and the test stage (presentation of the CS alone) (Bouton, 2002; Boschen, 2009). For example, if acquisition and extinction of fear occurs in context A and B, respectively, then fear response in test phase is usually less intense when the animal is tested in context B than in context A and C. That is, the animal would easily retrieve extinction memory if test occurs in the extinction context (i.e., Context B), while extinction memory is hard to be retrieved when the animal is tested in a novel (C) or in the fear acquisition (A) context. In animal studies, fear memory renewal is defined as the freezing increase during the “different” (e.g., ABC) compared to the “same” (e.g., ABB) context in the test (Wang et al., 2016).

Accumulating evidence shows that mere presence of conspecific partner, whether familiar or unfamiliar, is able to reduce one’s stress-related response in both human and animal subjects (Fontana et al., 1999; Hennessy et al., 2000). This phenomenon is called social buffering, which has been extensively observed across species, including rodents, sheep, pigs, non-human primates and also humans (Hennessy et al., 2000, 2009; Kikusui et al., 2006). For example, rodent studies indicate that the locomotor activity decreases when fear conditioned rats are subject to the CS alone, but the activity increases when they are accompanied by another non-fearful rat (Davitz and Mason, 1955). It was found that the rats’ freezing to contextual stimulus conditioned with shock was blocked by the presence of a partner (Kiyokawa et al., 2004). Furthermore, the freezing, and corticosterone level both decreased in the presence of a conspecific to fear-conditioned rats (Kiyokawa et al., 2014a), and it was reported that the presence of a conspecific animal suppresses CS-induced activation of amygdala (Fuzzo et al., 2015). These evidence indicates that social company is effective in reducing conditioned fear in rats.

For human studies, the psychotherapists require PTSD patients and their family members to receive therapeutic training together, in order to optimize the intervention effects for PTSD symptoms (Glynn, 1999; Sautter et al., 2009; Monson and Fredman, 2012). This implies that social company may disrupt the fear renewal in patients with PTSD. Therefore, we hypothesize that social company may play a critical role in suppressing fear renewal when an animal model is used. In a recent study close to this theme, Mikami et al. (2016) investigated how social company alters the effect of extinction training on fear retention in rats. The rats were firstly subjected to a fear conditioning procedure, and then experienced extinction procedure with or without social company. The results showed that social company enhanced the extinction effect in suppressing fear retention in ACC but not in ABC model (Mikami et al., 2016). However, the research purpose of this work determines that social company should be given to the extinction rather than to the test stage. To our knowledge, currently no study has examined the social buffering effect on fear renewal, by varying the time points to deliver company. Specifically, it is important to know when to offer social company may generate an optimal suppression of fear renewal. On the other hand, as the rehabilitated patients may experience various new situations after leaving a specific therapeutic situation, it is impossible to give social support in every new situation. In this regard, it is important to examine whether social company given to the extinction stage could reduce fear renewal in a novel context. Specifically, it requires examination whether social company given to the extinction stage may replace the fear suppression effect of social company offered to the test stage.

To address this issue, the present study used both ABC and ACC model to isolate a fear renewal effect. ACC, instead of ABB, is used in order to equate the test context during both conditions. Consequently, differential freezing among the ABC and ACC model is not attributable to physical differences in the test context, as all tests are conducted in an identical context. ABC model is used due to its close resemblance to the situation of fear memory renewal of PTSD patients in real life, in that novel contexts outside of the extinction one are common and the original context for fear acquisition is difficult to replicate (Balooch et al., 2012). We used two experiments to investigate social buffering effect on fear memory renewal. In the first experiment, all conditioned subjects underwent extinction individually and we manipulated social company at the test stage. We hypothesize that social company at the test stage could suppress the subjects’ fear renewal in the ABC model. In the second experiment, all the conditioned subjects underwent extinction with a conspecific animal, to examine how social company at extinction may alter the strength of fear renewal and its modulation by social company in the test. Based on the abundant evidence for context-dependent learning and social buffering phenomenon, we predict that animals in the test would exhibit higher fear response (i.e., more freezing) in ABC than in ACC model; while social company may disrupt the fear renewal effect, irrespective of the stage to offer social company.

## Materials and Methods

### Subjects and Housing

Both experiments were conducted in strict accordance with the recommendations of “Regulations for the Administration of Affairs Concerning Experimental Animals,” the State Science and Technology Commission, China. All animal procedures were approved by the animal care and use committee at Southwest University, China. 64 male Sprague-Dawley rats (180–220 g, postnatal age: about 60 days) including 52 subjects and 12 partners were purchased from the Institute of Traditional Medicine, Chongqing, China. Animals were housed in pairs in transparent cages (47 cm × 32 cm × 21cm) with corn-cob granule for bedding in a room maintained on a 12-h light/dark cycle (lights on at 8:00 a.m.) and were allowed to freely access food and water in their home cages. In each cage, two rats were assigned to be either subject or partner (i.e., rats placed with the subject during extinction or test), which ensured unfamiliarity between the subject and the accompanying rats. All the accompanying rats were manipulated to be unfamiliar to the subjects in this study, in order to control for the possible amplification of social buffering effect by familiarity (Hennessy et al., 2000; Kiyokawa et al., 2014b). All rats were handled (5 min per rat per day) for 5 days to habituate to the experimenters. All behavioral procedures were performed at 8:30 a.m.

### Apparatus

Four identical and standard rodent conditioning observation chambers (30.1 cm × 24.7 cm × 23.3 cm; Clever System Inc., Vienna, VA, United States) were used in the experiments. The chambers consisted of aluminum (side walls) and Plexiglas (rear wall, ceiling, and detachable front door). Digital video cameras were mounted on top of each chamber to videotape rats’ behavior. A speaker was mounted outside the wall of each chamber and was used for delivery of white noise.

Context A was a semi-circular chamber made by placing a curved plastic board into the standard rodent conditioning chamber. The floor of each chamber consisted of 18 stainless steel rods (5 mm diameter) spaced 1.6 cm apart. The rods were wired to a shock source for delivery of footshocks. The white light within the chambers was provided for illumination and the experimental room was dark. Stainless steel pans were placed underneath the grid floors and the chambers were sprayed with 2% acetic acid before animals were placed into it.

Context B was an opaque cask (25 cm diameter and 23 cm height) with a transparent cover inside the standard rodent conditioning chamber. A hole (10 cm diameter) was designed in the cover, to facilitate the rats’ breathing. Context B did not have stainless steel rods on the floor. To protect subjects’ activity or freezing from being disturbed by partners when subjects received the white noise (CS), all casks were approximately bisected into 250-cm^2^ with a transparent plastic PVC partition. There are 9 holes (2 cm diameter) in transparent plastic board for subject and partner to communicate by visual, olfactory or restricted tactile modalities (Kiyokawa et al., 2009). Olfactory communications were allowed because the main olfactory system, which underlies the processing of conspecific olfactory signals, has been verified to mediate the social buffering effect in rats (Kiyokawa et al., 2012). The partner was placed on one side of the cask before subject entered in. The white light within the chambers was provided for illumination and the experimental room was bright. The chambers were sprayed and cleaned with 75% ethyl alcohol before animals were placed into it.

Context C was formed by the standard rodent conditioning chamber, with the floor of each consisting of 16 stainless steel rods alternate in thickness. To protect subjects’ activity or freezing from being disturbed by the partners when subjects received the white noise (CS), all chambers were bisected into approximately 360-cm^2^ with a transparent plastic PVC partition. Similarly, there are 9 holes (2 cm diameter) in transparent plastic board for subject and partner to communicate by visual, olfactory or restricted tactile modalities. The partner was placed on one side of the box before subject entered in it. The white light within the chamber was provided for illumination and the experimental room was bright. The chambers were sprayed and cleaned with 2% isoamyl alcohol before animals were placed into it.

### Experiment Design

In Experiment 1, there were three experimental phases: fear conditioning, extinction, and test. For fear conditioning (Day 1), each time a maximum of 4 subjects were transported to context A in pairs in their home cages, which were covered with a white trash bag. All the subjects received auditory fear conditioning which consisted of 4 trials of 30 s, 80 dB, white-noise (conditioned stimulus, CS) co-terminating with a 1 s, 0.5 mA foot shock (1 min intertrial interval, unconditioned stimulus, US). The white noise started 3 min after subjects were placed in context A. After the final trial was finished, the animals were immediately returned to the homecage and the shock grids and floor trays were cleaned. Partners stayed in the feeding room with no foot shock.

**FIGURE 1:**
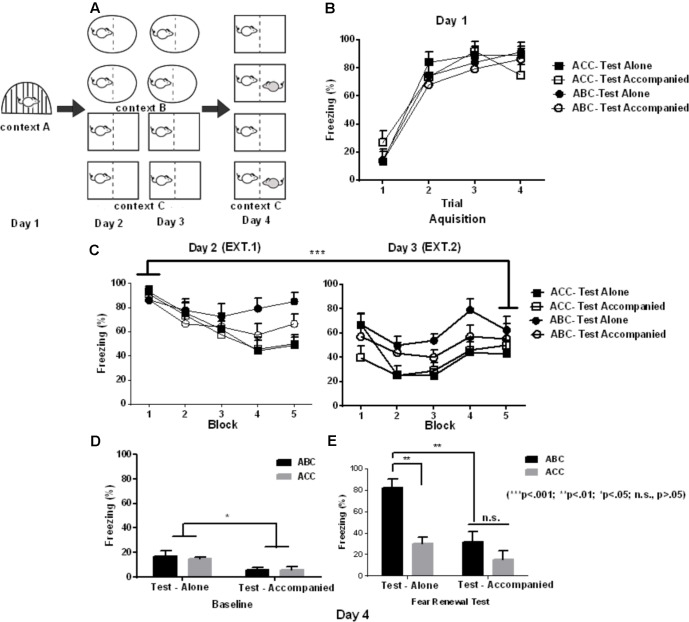
Effects of social buffering on fear memory renewal in Experiment 1. **(A)** Schematic illustration of Experimental design (shape denotes context), yielding a total of 4 groups in a 2 ^∗^ 2 design (context ^∗^ company): ACC-Test alone (*n* = 8), ACC-Test accompanied (*n* = 7), ABC-Test alone (*n* = 7), and ABC-Test accompanied (*n* = 6). **(B)** Freezing in fear acquisition (Day 1) varying with the trial of shock in each group (CS-US). **(C)** Freezing in extinction averaged in blocks of 4 trials (4 CS) in Day 2 and 3. **(D)** Baseline freezing in the 180-s baseline period prior to CS onset. **(E)** Freezing in the Same (ACC) or updated (ABC) context in the first test block. It can be observed that the robust fear renewal effect was blocked by partner presentation. Error bar denotes ± SEM.

On Day 2 and Day 3, subjects were transported to context B in pairs in their homecages that were covered with a yellow trash bag in the ABC condition (**Figure 1A**). In the ACC condition, subjects were transported to context C in pairs in their homecages covered with a black trash bag. All the subjects, irrespective of condition, received 20 white-noise (30 s, 80 dB) in the absence of footshock, beginning 3 min after placement in their respective context without social company. After the final trial was finished, the animals were immediately returned to the homecage.

On Day 4, subjects were transported to context C in pairs in their homecages that were covered with a black trash bag. All the subjects received renewal test with or without partner. The accompanying rats were unfamiliar to the subjects. The subject and the accompanying rats were neither housed together nor had any physical contacts before experiment. The test consisted of 6 trials of white-noise (30 s, 80 dB) in the absence of footshock, beginning 3 min after placement in the context C. The animals were returned to the homecage immediately after the final trial was finished. The test occurred either in the extinction context (ACC) or in a novel context (ABC). This yields a total of 4 groups in a 2 ^∗^ 2 design (context ^∗^ company): ACC-Test alone (*n* = 8), ACC-Test accompanied (*n* = 7), ABC-Test alone (*n* = 7), and ABC-Test accompanied (*n* = 6).

**FIGURE 2:**
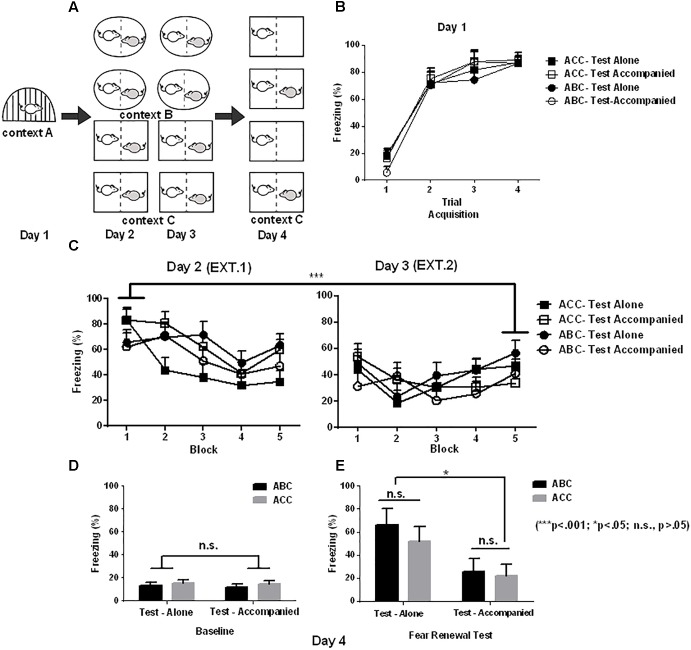
Effects of social buffering on fear memory renewal in Experiment 2. **(A)** Schematic illustration of Experimental design (shape denotes context), yielding a total of 4 groups in a 2 ^∗^ 2 design (context ^∗^ company): ACC-Test alone (*n* = 6), ACC-Test accompanied (*n* = 7), ABC-Test alone (*n* = 6), and ABC-Test accompanied (*n* = 5). **(B)** Freezing in fear acquisition (Day 1) varying with the trial of shock in each group (CS-US). **(C)** Freezing in extinction averaged in blocks of 4 trials (4 CS) in Day 2 and 3. **(D)** Baseline freezing in the 180-s baseline period prior to CS onset. **(E)** Freezing in the Same (ACC) or updated (ABC) context in the first test block. It can be observed that the fear renewal effect, indexed by ABC-ACC difference in freezing, was no longer significant in the test after accompanied extinction. Error bar denotes ± SEM.

Experiment 2 had the same procedure as described above with the exception that all the subjects underwent extinction training in the presence of a naïve unconditioned partner (with social company). It also yields a total of 4 groups in a 2 ^∗^ 2 design (context ^∗^ company): ACC-Test alone (*n* = 6), ACC-Test accompanied (*n* = 7), ABC-Test alone (*n* = 6), and ABC-Test accompanied (*n* = 5) (**Figure 2A**).

### Data Collection and Analysis

Freezing was defined as an immobile posture and was measured during the 30-s period after the onset of each CS (trial). We calculated the percentage of time spent in a freezing posture with respect to the 30-s period in every CS trial during the acquisition phase. All of the freezing was recorded and analyzed with digital video cameras by using commercially available software (Freezescan, Clever System Inc., Vienna, VA, United States). The averaged freezing of every 4 CS was calculated as a block for the extinction and the averaged freezing of every 2 CS as a block for the test. Freezing during fear conditioning was analyzed via two-way repeated ANOVA with Group as the between-subjects factor and Trial as the within subjects factor. After this, animals were equally split into groups (ABC/ACC and test-alone/accompanied) based on their freezing level in the acquisition phase, ensuring a similar level of freezing before extinction. Freezing during extinction was analyzed via a three-way mixed-design ANOVA with Time (2 levels, the first block vs. the last block) as a repeated factor while Company and Context as two between-subjects factors. Baseline freezing in the test was analyzed via a two-way ANOVA with Company and Context as between-subjects factors. Baseline freezing was measured in the first 3 min before the onset of the first CS during test (Kiyokawa et al., 2007). Freezing in the test was analyzed via a three-way mixed-design ANOVA with Block (3 levels) as a repeated factor while Company (2 levels) and Context (2 levels; ABC vs. ACC) as two between-subjects factors. *Post hoc* comparisons with Bonferroni method were performed after a significant omnibus F-ratio was detected. Student’s t-test was used to investigate the difference between EXT.1 ACC-test alone and EXT.2 ACC-test alone groups. The first block of two trials during test was chosen as the key indicator of fear renewal. The sample size of both experiments was determined by the principle (E = Total number of animals - Total number of groups ≥ 20) recommended by Charan and Kantharia (2013). Accordingly, 28 rats were used for Experiment 1 (*E* = 24) and 24 rats (*E* = 20) were used for Experiment 2, in order to obtain an *E* value no less than 20. *Post hoc* power analysis via G^∗^power indicated that the current sample size of two experiment (5–8 rats for each condition) is sufficient to obtain a reliable statistical power for the key results reported in this study (all observed powers > 0.9). Statistical significance was accepted at *p* < 0.05, two-tailed.

## Results

In Experiment 1, **Figure 1B** shows that the subjects’ freezing during acquisition was affected by the trial [context A; *F*(3,72) = 67.82; *p* < 0.001; $\eta_{p}^{2}$ = 0.739], with the percent of freezing significantly increased in the Trial 2–4 than in Trial 1. There is no significant main effect of group [*F*(3,24) = 0.70; *p* = 0.56] or significant interaction effect between group and trial [*F*(9,72) = 1.56; *p* = 0.18].

Freezing averaged in each of the 10 blocks during extinction is shown in **Figure 1C**. The data from the first and the last block were used for statistical analysis. A 2 (Context) ^∗^ 2 (Company) ^∗^ 2 (Block) mixed-design ANOVA of freezing during extinction revealed a significant main effect of Block [*F*(1,24) = 34.46; *p* < 0.001; $\eta_{p}^{2}$ = 0.58), with the percent of freezing significantly decreased in the last than in the first block. There were no significant main effects of Context [*F*(1,24) = 0.42; *p* = 0.52] and Company [*F*(1,24) = 0.03; *p* = 0.85]. There was no significant Context and Block interaction [*F*(1,24) = 2.5; *p* = 0.12], Context by Company interaction [*F*(1,24) = 0.19; *p* = 0.67], Context by Block interaction [*F*(1,24) = 2.5; *p* = 0.12], or Context by Block by Company [*F*(1,24) = 0.24; *p* = 0.63]. These data suggest that the extinction was successful, and the extinction effect was similar across the four samples before the test.

After extinction, rats were tested for fear renewal in the same context as extinction (“ACC” design) or shifted out of the extinction context (“ABC” renewal). **Figure 1D** shows the freezing in the baseline. A 2 (Company) ^∗^ 2 (Context) ANOVA of the baseline freezing showed a main effect of Company [*F*(1,24) = 9.55; *p* = 0.005; $\eta_{p}^{2}$ = 0.285], while there was no significant main effect of Context [*F*(1,24) = 0.00; *p* = 0.97] or significant interaction between Company and Context [*F*(1,24) = 0.42; *p* = 0.52]. This suggests that the accompanied groups had less freezing than the alone groups during the baseline.

**Figure 1E** shows the freezing in the first block of the test, as the first test block has proven to exhibit the strongest fear renewal effect (Corcoran et al., 2005). For the freezing analysis in the test stage, there were significant main effects of Block [*F*(2,48) = 9.20; *p* < 0.001; $\eta_{p}^{2}$ = 0.277] and Context [*F*(1,24) = 9.16; *p* = 0.006; $\eta_{p}^{2}$ = 0.276], and a significant Block by Context interaction [*F*(2,48) = 9.33; *p* < 0.001; $\eta_{p}^{2}$ = 0.280]. *Post hoc* comparisons indicates that subjects in ABC model showed a significantly higher freezing than subjects in ACC model from block 1 to block 3 (all *p* < 0.05), while this effect was most pronounced in block 1 (*p* < 0.001). This indicates that ABC model induced a robust fear renewal effect. There was a significant main effect of Company, with the accompanied subjects showing reduced freezing compared to the alone subjects [*F*(1,24) = 7.81; *p* = 0.01].

More importantly, there was a significant interaction between Company and Context [*F*(1,24) = 5.42; *p* = 0.03; $\eta_{p}^{2}$ = 0.184]. The *post hoc* comparisons revealed that the difference between accompanied and alone conditions was not significant in ACC model (*p* = 0.74); while the accompanied group showed significantly less freezing compared to the alone group in ABC model (*p* = 0.002). On the other hand, the alone subjects showed a higher percent of freezing in the ABC vs. ACC conditions (*p* = 0.002), while the accompanied subjects showed a similar level of freezing across ABC and ACC conditions (*p* = 0.64). The above company by context interaction was unaffected by block, shown by the non-significant company ^∗^ context ^∗^ block interaction [*F*(2,48) = 1.47; *p* = 0.24].

In Experiment 2, **Figure 2B** shows that the subjects’ freezing during acquisition was more pronounced in the Trial 2–4 than in Trial 1 [context A; *F*(3,60) = 114.60; *p* < 0.001; $\eta_{p}^{2}$ = 0.851]. There was no significant main effect of Group [*F*(3,20) = 0.19; *p* = 0.90] or significant interaction between Group and Trial [*F*(9,60) = 0.61; *p* = 0.73].

The averaged freezing from block 1 to 10 in the extinction is depicted in **Figure 2C**. A 2 (Context) ^∗^ 2 (Company) ^∗^ 2 (Block) mixed-design ANOVA of freezing during extinction revealed a significant main effect of Block [*F*(1,20) = 13.92; *p* < 0.001; $\eta_{p}^{2}$ = 0.41], with the freezing rate significantly decreased in the last than in the first block. The main effects of Context [*F*(1,20) = 0.78; *p* = 0.39] and Company [*F*(1,20) = 0.17; *p* = 0.19] were non-significant. There was no significant Context and Block interaction [*F*(1,20) = 3.1; *p* = 0.09], Context by Company interaction [*F*(1,20) = 0.06; *p* = 0.81], Company by Block interaction [*F*(1,20) = 0.63; *p* = 0.44], and no significant Context by Block by Company interaction [*F*(1,20) = 0.001; *p* = 0.98]. These results suggest that the extinction was successful, and the extinction effect was similar across the four samples before the test.

After extinction, rats were tested for fear renewal in the same context as extinction (“ACC”) or shifted out of the extinction context (“ABC”). **Figure 2D** shows the freezing in the baseline. The 2 (Company) ^∗^ 2 (Context) ANOVA of baseline freezing showed no main effect of Company [*F*(1,20) = 0.15; *P* = 0.70] or Context [*F*(1,20) = 0.87; *P* = 0.36]. Also, there was no significant interaction between Company and Group [*F*(1,20) = 0.001; *P* = 0.97]. This suggests that distinct from that in Experiment 1, the baseline freezing in Experiment 2 was similar across the four groups when the extinction was performed with company (**Figure 2D**).

**Figure 2E** shows the freezing data for the first test block. For the freezing analysis in the test stage, we observed significant main effects of Company [*F*(1,20) = 4.428; *p* < 0.05; $\eta_{p}^{2}$ = 0.181] and Block [*F*(2,40) = 7.14; *p* < 0.05; $\eta_{p}^{2}$ = 0.263], and a significant Company by Block interaction [*F*(2,40) = 4.7; *p* = 0.025; $\eta_{p}^{2}$ = 0.191]. The accompanied groups exhibited less freezing than the alone groups, and this effect was most pronounced in the first block (*p* = 0.01). On the other hand, the accompanied groups showed similar freezing from the first to the third block (*p* = 0.76), while the alone groups showed less freezing from the first to the third block (*p* < 0.05).

There was no significant main effect of Context [*F*(1,20) = 0.001; *p* = 0.97], and no significant Company by Context [*F*(1,20) = 0.02; *p* = 0.89], or Company by Context by Block interaction [*F*(2,40) = 0.50; *p* = 0.56]. These data suggest that social company offered to the extinction stage disrupted the robust fear memory renewal effect as observed in Experiment 1.

In order to examine whether test alone in ACC of Experiment 2 induces freezing due to removal of partner in test compared to extinction context, we compared the test-alone freezing of ACC model between Experiment 1 and Experiment 2 by the Student’s *t*-test. The results showed no significant differences between Experiment 1 and Experiment 2 [*t*(7) = -1.484, *p* = 0.183]. Thus, removal of partner in the test-alone condition of ACC model in Experiment 2 did not significantly induce fear renewal.

## Discussion

Giving social company at different stage in two experiments, the current study focuses on how social company modulates fear memory renewal elicited by contextual updating. Fear memory renewal was operationally defined by the contrast of freezing rate between ABC and ACC model. Experiment 1 manipulated social company at the test rather than the extinction stage, to examine how social company in the test phase alone may alter fear memory renewal. The results firstly showed that rats exhibited more freezing in ABC than in ACC model, which is consistent with previous finding of robust fear memory renewal in the ABC model (Wang et al., 2016). Then, we observed no significant freezing differences in the test between accompanied and alone conditions in ACC model. This is consistent with the reports of Nowak et al. (2013), which observed a similar freezing for the mice with the presence of naïve associate and those without associate in the ABB paradigm (equivalent to the current ACC paradigm) (Nowak et al., 2013). It is worth noting that the authors of this work used an experimental design allowing the mice to see, hear and smell the neighbor, but not to contact conspecific physically. The authors thought that absence of direct physical contact impeded the interaction between the animals, thereby they did not observe a significant difference in the levels of fear response between the mice with and without naïve associates. In our study the visual, auditory and limited olfactory contacts were allowed, to optimize the social buffering effect. In this regard, we infer that the lack of social buffering effect in ACC model should not be due to the insufficient provision of social company. Instead, this is most likely due to the floor effect of freezing, as the rats were tested in the same context as in the extinction, which facilitates the retrieval of the fear unlearning memories (Myers and Davis, 2007).

Importantly, the accompanied group showed significantly less freezing than alone group during test in ABC model, which implies that social company during test could suppress fear memory renewal from context updating. This is consistent with prior reports that the presence of a conspecific, whether familiar or unfamiliar to the subject, ameliorated conditioned fear responses in behavioral (e.g., freezing) and physiological (e.g., corticosterone) measures when the animal was tested after a fear conditioning procedure (Kiyokawa et al., 2007, 2014a; Ishii et al., 2016). However, these studies did not design an extinction phase, thus unable to allow for the observation of a fear renewal effect and how it varies with social company. Thus, the current study extends previous studies by showing that social company does not only ameliorate fear responses evoked by the CS, but is also able to mitigate the fear renewal in response to the CS occurring in a novel context outside of the extinction one.

As described above, Experiment 1 showed a clear social buffering effect on fear memory renewal when social company was offered to the test stage. However, the practical implication of this finding is limited. This is due to the fact that the rehabilitated patients in the real-life settings may experience various new situations after leaving a specific therapeutic situation, as stated above. This prompted Experiment 2 that focused on whether social company given to the extinction stage may suppress the fear renewal effect in a novel context. The results showed that the subjects’ freezing in the test stage was no longer significantly different between ABC and ACC model, implying that social company given to the extinction stage disrupted the robust fear memory renewal effect.

However, one may question that in Experiment 2, the lack of higher freezing from ACC to ABC model in the test-alone condition does not necessarily reflect social buffering effect due to providing company to the extinction stage. Instead, it may reflect enhanced freezing in the ACC model due to removal of company from the extinction to the test stage. This possibility should be considered as prior studies indicated that context includes not only external physical environment but also internal cognitive context (Bouton et al., 2006; Maren et al., 2013). However, this may not contaminate our conclusion as our comparison of test-alone freezing in ACC model showed no significant differences from Experiment 1 to Experiment 2. This suggests that partner removal did not significantly induce fear renewal, as freezing was similar no matter whether the test-alone context was constituted by partner removal or by the lack of partner all the time. This inference was further confirmed by our finding that, adding a partner to the test context did not alter the freezing rate when comparing accompanied to alone condition in the ACC model of Experiment 1 (see **Figure 1E**). It is still necessary to conduct confirmatory research manipulating Extinction Company in parallel with Context in the test-alone condition with a larger sample size. This allows a more rigid statistical examination of whether removal of partner from extinction to test indeed elicits no effect of fear renewal.

Another issue worth noting is that the current findings appear discrepant with those by Mikami et al. (2016). In this work, the authors manipulated the presence of social company, that is, providing a conspecific male rat or not to the subject, to investigate how the effect of extinction training on conditioned fear varies with social company. The results showed that the extinction training suppressed freezing elicited by the CS in the ABB paradigm (i.e., test and extinction context was the same) when social company was available. However, the above extinction effect vanished when social company is absent, or when social company is provided but the subject was tested in a novel context (Mikami et al., 2016). Thus, comparing these data with the current findings may leave an inconsistent impression, in that we observed social company given to the extinction stage disrupted fear renewal when the subject was tested in a novel context. However, the current study differs from this work in two important ways. Firstly, the extinction training of the two studies differs in both duration and intensities. The extinction training of Mikami et al. (2016) consisted of 24 trials of CS delivered in 1 day, while the current extinction procedure consisted of 40 trials of CS divided by 2 days.

More importantly, driven by the research purpose of how the effects of extinction training on fear response may be moderated by social company, Mikami et al. (2016) focused on whether the freezing differences between extinction and non-extinction in the recall test vary with social company and contextual updating. However, the current study focuses on how social company may modulate the effect of fear memory renewal elicited by context updating. Driven by this purpose, the current study did not manipulate extinction. Instead, we let all the subjects receive a 2-day extinction, in order to manipulate the context in the test (same/different). Accordingly, we mainly tested how ABC-ACC differences in freezing varied as a function of social company (accompanied vs. alone). In this regard, our observation that social company eliminated the fear renewal effect during ABC vs. ACC contrast, is not incompatible with the finding of Mikami and colleagues that 24 trials of accompanied extinction in 1 day mitigated the animals’ fear-conditioned response in ABB but not in ABC model.

Prior studies in protection from extinction showed that pairing an inhibitory CS (a predictor for the absence of the US) with a fear-eliciting cue during extinction impedes extinction, leading to a return of fear response when the inhibitory CS is removed in the test (Rescorla, 2003; Lovibond et al., 2009). In Experiment 2, we observed that the presence of social conspecific during extinction inhibited the animal’s fear renewal effect in the test stage, though the animal was tested alone and in a novel context. This result is not incompatible with the “protection from extinction” studies. Firstly, the role of an inhibitory CS in these studies is predicting the absence of the US. Thus, it is unsurprising that the individual’s expectancy of the US would appear again if the inhibitory CS is removed. By contrast, the role of social company is to provide social supports and relieve the stress, instead of predicting environmental safety (Kikusui et al., 2006). Secondly, the inhibitory CS works based on a long-term training of subjects in the laboratory, to obtain a safety prediction. By contrast, social company by presenting conspecific may inherently links to safety information (Hornstein et al., 2016).

To the best of our knowledge, previous studies have provided social company at different time points of fear conditioning, to examine how social company influences fear response, such as pre-fear conditioning (Guzman et al., 2009) or in fear acquisition (Lee and Noh, 2016), extinction (Mikami et al., 2016), or test (Kiyokawa et al., 2014a). However, little research has examined the role of social company in fear renewal by employing the ACC and ABC paradigms simultaneously. The benefit of using two models simultaneously is that the differential freezing between the ABC and ACC model is not attributable to physical differences in the test context because all tests are conducted in an identical context with the same CS. The current study extends prior studies by exploring the suppression of fear renewal by social company and how to maximize the suppression effect by manipulating the time points to offer company. Taken together, the two studies not only provide evidence that social company is able to mitigate fear renewal, but also suggests that social company given at the extinction stage generates an optimal suppression of fear renewal. Though massive extinction treatment has been suggested to attenuate the fear renewal effect (Denniston et al., 2003), it is more practical and economical to provide social company during treatment in the limited time of the therapist. Future studies need to examine the neural pathway subserving social buffering effect on fear renewal, in addition to the current understanding of neural pathway of social buffering (Kiyokawa et al., 2012). Another point worth noting is that the accompanying rats used in our study were all unfamiliar to the subjects, for isolating a unique effect of social buffering. A handful of studies have shown that familiar conspecifics elicit better social buffering effects compared to unfamiliar ones (Hennessy et al., 2000; Kiyokawa et al., 2014b). In this regard, future studies should consider using familiar partner to explore social buffering effects on clinical PTSD, in order to seek an optimal intervention effect.

## Author Contributions

JY, MY, and XW performed the experiments. WC helped in experimental design. JY and MY analyzed the data. JY, MY, and YX wrote the paper.

## Conflict of Interest Statement

The authors declare that the research was conducted in the absence of any commercial or financial relationships that could be construed as a potential conflict of interest.
